# Determinants of AI Adoption in Healthcare: Insights From a Unified Theory of Acceptance and Use of Technology (UTAUT) Study Among Doctors and Nurses in a Tertiary Care Hospital in North India

**DOI:** 10.7759/cureus.104086

**Published:** 2026-02-22

**Authors:** Sushila Kataria, Rhea Aggarwal, Anuja Ashok Ardhapure, Vinod Krishnankutty, Pooja Sharma, Adarsh Keshari, Pranshul Kataria

**Affiliations:** 1 Internal Medicine, Medanta the Medicity, Gurugram, IND; 2 Nursing, Medanta the Medicity, Gurugram, IND; 3 Obstetrics and Gynecology, APAR Health, Gurugram, IND; 4 Research, APAR Health, Gurugram, IND; 5 School of Medicine, Newcastle University, Newcastle, GBR

**Keywords:** artificial intelligence in healthcare, clinical decision support systems (cdss), digital health adoption, digital health / health informatics, healthcare professionals', technology acceptance, unified theory of acceptance and use of technology (utaut)

## Abstract

Background

Artificial Intelligence tools are increasingly entering clinical practice, yet their adoption depends on how healthcare professionals perceive, experience, and intend to use them. Understanding factors associated with adoption is critical for designing targeted AI education.

Objectives

To assess acceptance of AI-based tools among doctors and nurses in a tertiary hospital in North India using a tool based on the Unified Theory of Acceptance and Use of Technology (UTAUT), and to identify early and late adopters based on prior AI exposure, training, and behavioral intention.

Methods

A cross-sectional mixed-methods study was conducted among 256 healthcare professionals (116 doctors, 140 nurses) at a multispecialty tertiary-care hospital. Prior to the administration of the questionnaire, an informative video introducing key concepts and applications of artificial intelligence in healthcare was shared with all participants to ensure a common baseline understanding. The structured UTAUT-based questionnaire captured demographics, AI exposure and training, perceived usefulness, accuracy, helpfulness, workload impact, behavioral intention, and barriers/enablers, along with open-ended questions. Quantitative analysis compared perceptions between those with and without prior AI experience, stratified by profession, and explored early adopters based on pre-specified predictor items. In an additional exploratory step, composite early-adopter definitions were constructed using AI exposure, formal training, tool use, and consistently high intention scores.

Results

Only about one-fifth of doctors (21.6%) and nurses (19.3%) reported prior AI experience in healthcare, and most of those with experience had used AI-enabled tools such as clinical decision support or drug-interaction checkers. Doctors reported the highest likelihood of using AI for drug-drug interactions, drug dosing and patient follow-up, whereas nurses rated AI as most useful for monitoring, documentation and nutrition support. Among doctors, prior AI experience was associated with a significantly higher likelihood of using AI for diagnosis, follow-up and triage, and with greater perceived helpfulness in triage. Across all participants, prior AI experience increased intention for daily clinical activities and clinical decision-making, although not for workflow tasks. When early adopters were defined broadly (any exposure, training or high intention), over 85% met criteria; however, under a strict definition requiring training, exposure, tool-use and uniformly high intention, only 3.5% qualified as AI champions, predominantly nurses. Trust, medico-legal concerns and limited awareness were the most frequently reported barriers by both; however, nurses more frequently expressed fear of job displacement. Training, institutional support and integration with electronic medical records were identified as key enablers.

Conclusions

Doctors and nurses showed different patterns of AI readiness. Doctors with prior AI exposure showed greater willingness to use AI, reflecting higher trust in AI for high- risk clinical decision support tasks. Nurses demonstrated high baseline acceptance, with less variation by prior AI use; however, formal training clearly distinguished early adopters. In conclusion, targeted, profession-specific training would serve as a key enabler to support effective and responsible AI adoption in healthcare.

## Introduction

Artificial Intelligence (AI) has emerged as a major driver of digital transformation in healthcare, with applications in diagnostics, clinical decision support, patient monitoring, and streamlining documentation [1-3]. Across clinical medicine, artificial intelligence has been associated with measurable improvements in diagnostic accuracy, therapeutic decision support, operational efficiency, and patient safety across diverse clinical domains [4]. Yet the success of AI implementation is not determined only by technical performance; it depends more so on the acceptance, trust, and behavioral intention of healthcare professionals who are expected to integrate these tools into routine practice [5,6].

The Unified Theory of Acceptance and Use of Technology (UTAUT) provides a robust framework to study technology adoption behavior [7]. It posits that performance expectancy, effort expectancy, social influence, and facilitating conditions shape behavioral intention and use. This model has been widely applied to digital health technologies but less frequently to AI in real-world clinical contexts, especially in low and middle-income settings [6,8]. In this study, UTAUT constructs were used to structure the assessment of AI acceptance, behavioral intention, and adoption readiness among healthcare professionals.

Existing literature highlights that while the awareness of AI in healthcare is rising, confidence and preparedness remain limited. Surveys of health professionals and clinicians in high-income settings report neutral or skeptical attitudes, driven by lack of trust, transparency, medico-legal uncertainty, and limited or no training [5,9]. They indicate common barriers: ethical ambiguity, unclear liability, and insufficient institutional support across regions, including the UK, the Middle East, and Asia [6,8]. At the same time, reports such as the Philips Future Health Index note that healthcare professionals see clear value in AI for administrative tasks, highlighting its potential for use, but feel underprepared for clinical integration [10].

Despite increasing availability of AI tools in Indian hospitals, there is limited empirical evidence on how doctors and nurses- two core professional groups- differ in their readiness to adopt AI and what predicts early versus late adoption. Understanding these patterns can be pivotal for designing tailored educational and institutional strategies, rather than treating “healthcare professionals” as a homogeneous group.

To address these gaps, we conducted this study, grounded in the UTAUT framework. The primary objective of this study was to assess acceptance of artificial intelligence-based tools among doctors and nurses in a tertiary care hospital using a Unified Theory of Acceptance and Use of Technology (UTAUT) based framework. Secondary objectives were to examine the association between prior exposure to artificial intelligence, formal training, and behavioral intention to use AI; to identify early and late adopters within each professional group; and to explore perceived barriers and enablers influencing adoption of AI in routine clinical practice.

## Materials and methods

Study design and setting

We conducted a cross-sectional study among doctors and nurses using the UTAUT framework. The study was based at a large multispecialty tertiary-care hospital (Medanta The Medicity, Gurugram, North India), and included participants from multiple clinical departments without restricting recruitment to a specific speciality. Prior to starting the questionnaire, an informative video about AI in healthcare, currently and its prospects, was shared with the participants.

Participants and sampling

A total of 256 healthcare professionals were recruited, comprising 116 doctors and 140 nurses. Inclusion criteria were: MBBS-qualified doctors or registered nurses working full-time, with at least one year of clinical experience, and willingness to provide informed consent. The initial sampling plan stratified both doctors and nurses by years of clinical experience (≤5, 5-10, 10-15, >15 years) to ensure representation across career stages.

Data collection tool

A structured questionnaire based on the Unified Theory of Acceptance and Use of Technology (UTAUT) framework was used for data collection. The UTAUT framework was originally proposed by Venkatesh et al. and is freely available for academic and research use. The questionnaire items were adapted from previously validated UTAUT-based instruments without the use of any proprietary scales or licensed tools [11]. The questionnaire captured demographic and professional characteristics, including age, gender, qualification, profession, years of clinical experience, and department. It also assessed prior exposure to artificial intelligence in healthcare, including past AI experience, formal AI training, and use of AI tools such as clinical decision support systems and general-purpose AI platforms. Core UTAUT constructs, performance expectancy, effort expectancy, social influence, and facilitating conditions, were measured using 20 items rated on a 5-point Likert scale, ranging from strongly disagree (1) to strongly agree (5).

The questionnaire was based on the Unified Theory of Acceptance and Use of Technology (UTAUT) framework originally proposed by Venkatesh et al. [10] and adapted from previously published UTAUT-based instruments [12]. All attitudinal, likelihood, and behavioral intention items were measured using a standard 5-point Likert scale, ranging from strongly disagree (1) to strongly agree (5) or very unlikely (1) to very likely (5), as appropriate. The full questionnaire with item wording is provided in the Appendices A-E. Internal consistency testing (Cronbach’s alpha) was not performed as the instrument comprised adapted UTAUT constructs used descriptively rather than as a composite scale. Future validation studies may assess scale reliability.

The use of a 5-point Likert scale is widely accepted in healthcare technology adoption research for measuring perceptions, attitudes, and intention to use digital tools [13]. All Likert-scale items used in this study were non-proprietary, freely available for academic research use, and did not involve any copyrighted or licensed instruments.

Perceived usefulness, accuracy and helpfulness of AI as separate questions

Perceived usefulness, accuracy, and helpfulness of artificial intelligence were assessed as separate questions. For doctors, these items covered the use of AI in diagnosis, drug dosing, drug-drug interaction checking, interpretation of test reports or imaging, patient follow-up, and triage. For nurses, corresponding items assessed the use of AI for patient monitoring, including alerts and prediction of deterioration in vital signs, documentation support, nutrition support, and robotic caregiving systems.

Workload impact was assessed using two items that examined participants’ perceptions of whether AI would increase workload or waste time versus decrease workload or save time. Voluntariness and behavioral intention were assessed using four items that captured the perceived voluntariness of AI use and the intention to use AI in daily clinical activities, clinical decision-making, and administrative tasks.

Barriers and enablers were assessed using multiple-response items addressing perceived barriers to AI adoption, including lack of awareness, fear of job loss, trust-related concerns, legal or ethical issues, and infrastructural limitations, as well as factors that would facilitate AI adoption, such as training, availability of accuracy data, institutional support, integration with electronic medical records, and peer use.

Open-ended questions were included to explore participants’ expectations, perceived positive or potential uses of AI, concerns related to its use, and the support needed for AI adoption in clinical practice. A brief section captured self-reported use of general-purpose artificial intelligence (AI) systems by participants, including ChatGPT (OpenAI), Gemini (Google DeepMind), Grok (xAI), and Perplexity AI. These tools were included solely as examples for participant self-reporting. The investigators did not provide, recommend, deploy, or use any AI tools during the conduct of the study. Open-ended responses were analyzed using simple thematic grouping to identify recurring perspectives on AI benefits, concerns, and implementation needs. Formal qualitative coding frameworks were not applied as the qualitative component was exploratory.

All perceived usefulness, accuracy, and helpfulness items were measured using the same 5-point Likert scale structure to ensure consistency across constructs [11,13].

Definition of early and late adopters

The questionnaire included a predefined set of predictor items to classify participants as early or late adopters of artificial intelligence. Predictors common to both doctors and nurses included the presence of formal AI or health-technology training, past experience with AI in healthcare settings, use of AI tools, and high behavioral intention to use AI in daily clinical activities, clinical decision-making, and administrative tasks, defined as Likert scale scores of 4 or 5.

In addition, profession-specific likelihood items were assessed. For doctors, early adoption was characterized by a high likelihood of using AI for diagnosis, drug dosing, drug-drug interaction checking, interpretation of test reports or imaging, patient follow-up, and triage, with high likelihood defined as scores of 4 or 5. For nurses, early adoption was characterized by a high likelihood of using AI for patient monitoring, including alerts and prediction of deterioration, documentation, nutrition support, and robotic caregiving systems, again defined as scores of 4 or 5.

Using these criteria, early adopters within each profession were described in two ways. In the predictor-based approach, doctors and nurses with prior experience of artificial intelligence who demonstrated statistically significantly higher likelihood ratings, defined as scores of 4 or 5, for tasks such as diagnosis, patient follow-up, and triage, were classified as early adopters relative to their peers without prior AI experience.

In an exploratory composite approach, participants were classified as broad early adopters if they met any one of the following criteria: formal training in AI or health technology, past experience with AI in healthcare, use of AI tools, or a high score of 4 or 5 on any likelihood or behavioral intention item. A stricter category of AI champions was defined as participants who met all criteria, including formal AI training, past AI experience, use of AI tools, and uniformly high scores of 4 or 5 across all relevant likelihood and behavioral intention items.

High behavioral intention was defined a priori as Likert scale scores of 4 or 5, consistent with prior UTAUT-based technology acceptance studies [11,13].

Statistical analysis

The analysis began with a profiling study of participants, doctors and nurses, based on demographic characteristics, educational background, years of professional experience, as well as AI exposure and training.

Perceived usefulness, accuracy, and helpfulness of AI, workload impact, along with behavioral intention, and barriers/enablers were summarised separately for doctors and nurses using frequency distributions across the 0-5 scoring scale. For each item, the mean score and the proportion of respondents assigning high scores (4 or 5) were calculated. Likert-scale responses were treated as ordinal data and summarized using frequencies, proportions, and mean scores, consistent with prior UTAUT-based healthcare adoption studies [11,13].

Subsequently, results were stratified according to participants’ prior experience with AI. Differences between groups were assessed for statistical significance using the chi-square test for comparisons of proportions and the independent samples t-test for comparisons of mean scores. A p-value of <0.05 was considered statistically significant. All statistical analyses were performed using IBM SPSS Statistics for Windows, Version 24.0 (IBM Corp., Armonk, NY, USA).

In the exploratory composite analysis, we calculated the prevalence of broad and strict early adopters overall, by profession, and by experience strata to inform educational implications. As analyses were exploratory and descriptive, adjustments for multiple comparisons were not applied, and findings are interpreted cautiously.

Ethics approval was obtained from the Institutional Ethics Committee of Medanta The Medicity, and consent was taken from all participants prior to administering the questionnaire.

## Results

Demographics

The demographic and professional characteristics of the participants are presented in Table 1. Of the 256 respondents, 116 (45.3%) were doctors and 140 (54.7%) were nurses. A total of 116 doctors participated in the study. The majority belonged to medical specialities (n=71; 61.2%), followed by surgical specialities (n=30; 25.9%). Critical care specialists accounted for 10 doctors (8.6%), while administrative roles formed a small proportion (n=5; 4.3%). Within the medical category, internal medicine constituted the predominant subgroup, with notable representation from cardiology, endocrinology, gastroenterology and others. Among surgical specialities, plastic surgery comprised the largest subgroup, followed by orthopedics and other related surgical specialities.

**Table 1:** Background Profile of Study Participants.

Variables		Profession		
		Doctor (n= 116)	Nurses (n = 140)	Total (n=256)
Age Group	21 - 25	4 (3.4%)	49 (35%)	53 (20.7%)
	26 - 30	31 (26.7%)	42 (30%)	73 (28.5%)
	31 - 35	21 (18.1%)	17 (12.1%)	38 (14.8%)
	36 - 40	14 (12.1%)	12 (8.6%)	26 (10.2%)
	41 - 45	13 (11.2%)	10 (7.1%)	23 (9%)
	46 - 50	14 (12.1%)	5 (3.6%)	19 (7.4%)
	> 50	19 (16.4%)	5 (3.6%)	24 (9.4%)
Gender	Male	71 (61.2%)	38 (27.1%)	109 (42.6%)
	Female	45 (38.8%)	102 (72.9%)	147 (57.4%)
Highest qualification	Graduate	26 (22.4%)	0 (0%)	26 (10.2%)
	Post-graduate	44 (37.9%)	0 (0%)	44 (17.2%)
	Super-specialist	46 (39.7%)	0 (0%)	46 (18%)
	Others	0 (0%)	140 (100%)	140 (54.7%)
Years of clinical experience	<5 Years	32 (27.6%)	74 (52.9%)	106 (41.4%)
	5-10 Years	24 (20.7%)	33 (23.6%)	57 (22.3%)
	11-15 Years	20 (17.2%)	12 (8.6%)	32 (12.5%)
	>15 Years	40 (34.5%)	21 (15%)	61 (23.8%)
Have you had past experience with AI in a healthcare setting?	Yes	25 (21.6%)	27 (19.3%)	52 (20.3%)
	No	91 (78.4%)	113 (80.7%)	204 (79.7%)
If yes, have you used AI tools (e.g., Clinical Decision Support Systems (CDSS), interaction checkers)?	Yes	21 (18.1%)	26 (18.6%)	47 (18.4%)

Among doctors, 20.7% had 5-10 years of clinical experience, 17.2% had 10-15 years, and 34.5% had more than 15 years of experience, as presented in Table 2.

**Table 2:** Distribution of Doctors by Clinical Speciality and Subspeciality

Main Category	Total n (%)	Top Subspecialties	n
Medical	71 (61.2%)	Internal Medicine*	dominant
		Cardiology	5
		Endocrinology	5
		Gastroenterology	3
		Cardiac Electrophysiology	2
		Pediatrics / Emergency	2 each
Surgical	30 (25.9%)	Plastic Surgery	9
		Orthopedics	4
		Urology	3
		Pediatric Surgery	3
		CTVS	2
Critical Care	10 (8.6%)	Critical Care (ICU)	8
		Neurocritical Care	1
		Anaesthesia + CC	1
Others (Administration)	5 (4.3%)	Medical Administration	5

Nurses were stratified into six major categories: Medical, Interventional, Outpatient Department (OPD), Surgical, Critical Care, and Others. Medical category included general ward/IPD nursing assignments. Interventional category included chemotherapy day-care, dialysis, cath-lab, radiology, oncology day-care, and endoscopy. OPD category included outpatient nursing roles such as the RWA clinic and OPD clinics. Surgical covered perioperative and OT staff. Critical Care category included ICU variants such as NICU, SICU, CCU, and stroke units. Others covered roles such as physiotherapy support and blood bank assignments.

A total of 140 nurses were included. The largest segment comprised medical/ward-based nurses (n=89, 63.6%), reflecting a predominantly inpatient workforce. This was followed by interventional/procedure nurses (n=20, 14.3%), including those in chemotherapy, dialysis, cath-lab, radiology and endoscopy services. OPD/clinic nurses accounted for 15 (10.7%), while critical care and surgical nurses represented 5 (3.6%) each, forming smaller proportions of the sample.

Among nurses, 52.9% had less than five years of clinical experience, 23.6% had 5-10 years, 8.6% had 10-15 years, and 15.0% had more than 15 years of experience, as shown in Table 3.

**Table 3:** Department Wise Distribution of Nurses. Data are descriptive and presented as number (percentage). No inferential analysis was performed.

Category	n	%
Medical/ward nurses	89	63.6%
Interventional/procedure	20	14.3%
OPD/clinic	15	10.7%
Other/admin	6	4.3%
Critical care	5	3.6%
Surgical/OT	5	3.6%

Both doctors and nurses were represented across all experience strata, ensuring coverage of early, mid-career and senior clinicians.

**Figure 1:**
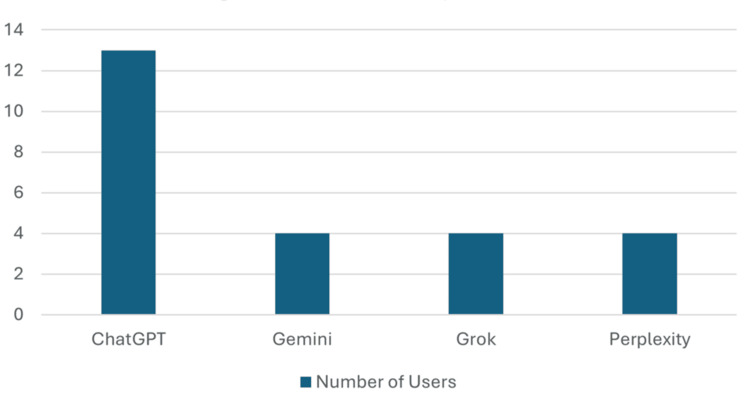
Prior AI Exposure. AI tools shown represent self-reported prior use by participants and were not provided, recommended, or mandated by the investigators. Perceptions and exposure were assessed using items adapted from non-proprietary Unified Theory of Acceptance and Use of Technology (UTAUT)–based instruments [11,12,13].

Figure 1 illustrates prior exposure to artificial intelligence among the study participants. Prior AI exposure in healthcare was limited but similar across professions: 21.6% of doctors and 19.3% of nurses reported past AI experience, and among these, approximately 90% in both groups had used AI-enabled tools such as CDSS or drug interaction checkers, beyond these, participants also reported informal use of general purpose systems such as ChatGPT, which was the most commonly reported (27.7%), followed by Gemini, Grok, and Perplexity (each 8.5%), reflecting early experimentation with large language models in this clinical workforce.

Table 4 presents overall perceptions of artificial intelligence usefulness, accuracy, and helpfulness among the study participants.

**Table 4:** Perceived Usefulness, Accuracy, and Helpfulness of AI Among Doctors and Nurses. Responses were collected using a 5-point Likert scale (1=very unlikely/not accurate/not helpful to 5=very likely/extremely accurate/extremely helpful), a standard approach for measuring attitudes and perceptions in health research [12]. Survey items were adapted from non-proprietary UTAUT-based technology acceptance instruments [11,12].

For Doctors (n=116)					
Perceived Usefulness of AI					
Questions	Very Unlikely	Unlikely	Neutral	Likely	Very Likely
Can AI be used in Diagnosis?	16 (13.8%)	23 (19.8%)	26 (22.4%)	30 (25.9%)	21 (18.1%)
Can AI be used for calculating drug dosing?	17 (14.7%)	17 (14.7%)	21 (18.1%)	28 (24.1%)	33 (28.4%)
Can AI be used in identifying drug-drug interactions?	12 (10.3%)	11 (9.5%)	23 (19.8%)	29 (25%)	41 (35.3%)
Can AI be used to help in analyzing test reports or imaging?	27 (23.3%)	18 (15.5%)	19 (16.4%)	22 (19%)	30 (25.9%)
Can AI be used to help with patient follow-up?	12 (10.3%)	12 (10.3%)	31 (26.7%)	33 (28.4%)	28 (24.1%)
Can AI be used in triage?	34 (29.3%)	22 (19%)	30 (25.9%)	10 (8.6%)	20 (17.2%)
Accuracy of AI					
Questions	Not Accurate at all	Slightly Accurate	Moderately Accurate	Very Accurate	Extremely Accurate
Can AI be used in diagnosing a patient?	11 (9.5%)	28 (24.1%)	46 (39.7%)	28 (24.1%)	3 (2.6%)
Can AI be used to identify appropriate drugs?	10 (8.6%)	20 (17.2%)	42 (36.2%)	32 (27.6%)	12 (10.3%)
Can AI be used to identify drug interactions?	5 (4.3%)	17 (14.7%)	24 (20.7%)	49 (42.2%)	21 (18.1%)
Can AI be used to analyse test reports or imaging?	16 (13.8%)	18 (15.5%)	38 (32.8%)	33 (28.4%)	11 (9.5%)
Helpfulness of AI					
Questions	Not Helpful at all	Slightly Helpful	Moderately Helpful	Very Helpful	Extremely Helpful
Can AI be in patient follow-up?	6 (5.2%)	11 (9.5%)	35 (30.2%)	47 (40.5%)	17 (14.7%)
Can AI be in triage?	21 (18.1%)	27 (23.3%)	37 (31.9%)	23 (19.8%)	8 (6.9%)
For Nurses (n = 140)					
Perceived usefulness of AI					
Questions	Very Unlikely	Unlikely	Neutral	Likely	Very Likely
Can AI be used in patient monitoring (i.e., to alert when vitals are low)?	27 (19.3%)	12 (8.6%)	24 (17.1%)	33 (23.6%)	44 (31.4%)
Can AI be used in patient monitoring (i.e., to predict worsening vital signs)?	22 (15.7%)	15 (10.7%)	30 (21.4%)	31 (22.1%)	42 (30%)
Can AI be used to help with documentation?	13 (9.3%)	14 (10%)	25 (17.9%)	35 (25%)	53 (37.9%)
Can AI be used to aid patient nutrition?	20 (14.3%)	18 (12.9%)	26 (18.6%)	34 (24.3%)	42 (30%)
Can AI be used to help with robotic nursing or caregiving systems?	38 (27.1%)	13 (9.3%)	29 (20.7%)	25 (17.9%)	35 (25%)
Accuracy of AI					
Questions	Not Accurate at all	Slightly Accurate	Moderately Accurate	Very Accurate	Extremely Accurate
Can AI be used in predicting the worsening of patient vital signs?	16 (11.4%)	14 (10%)	43 (30.7%)	36 (25.7%)	31 (22.1%)
Can AI be used in alerting to low vital signs?	18 (12.9%)	11 (7.9%)	37 (26.4%)	44 (31.4%)	30 (21.4%)
Helpfulness of AI					
Questions	Not Helpful at all	Slightly Helpful	Moderately Helpful	Very Helpful	Extremely Helpful
Can AI help with documentation?	14 (10%)	9 (6.4%)	24 (17.1%)	50 (35.7%)	43 (30.7%)
Can AI be with patient nutrition?	16 (11.4%)	14 (10%)	33 (23.6%)	47 (33.6%)	30 (21.4%)
Can AI help in the maintenance of patient mental health?	17 (12.1%)	20 (14.3%)	42 (30%)	35 (25%)	26 (18.6%)
How helpful do you think robotic nursing or caregiving systems would be?	33 (23.6%)	18 (12.9%)	30 (21.4%)	32 (22.9%)	27 (19.3%)

**Figure 2:**
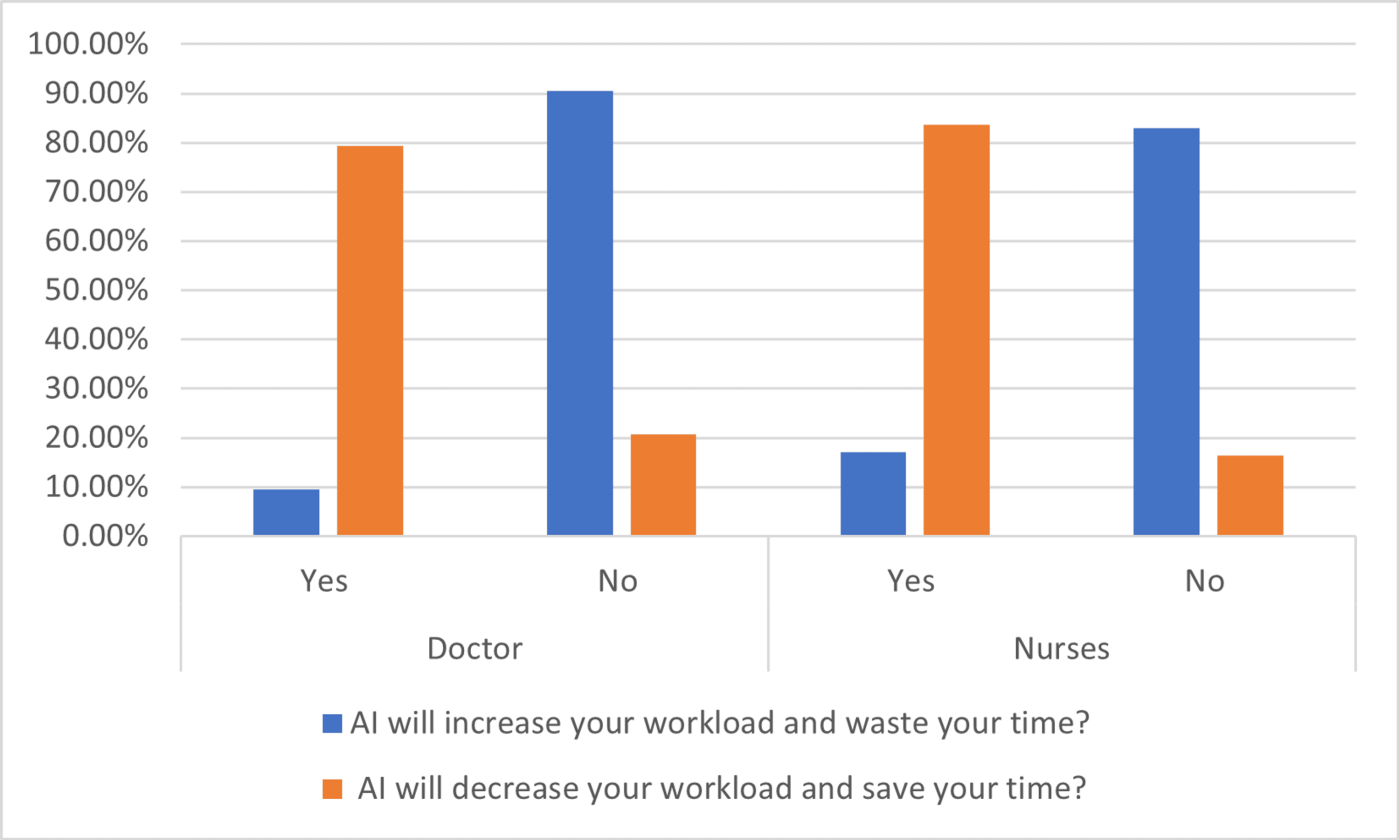
Workload Impact of Artificial Intelligence. Participants’ perceptions of AI-related workload impact were assessed using Likert-scale items adapted from UTAUT and extended technology acceptance frameworks, focusing on perceived efficiency, time savings, and burden reduction [11,12,13].

Figure 2 illustrates doctors’ and nurses’ perceptions of artificial intelligence-related workload impact

Among doctors, mean likelihood scores were highest for drug-related tasks, including drug-drug interaction checking and drug dosing, with a larger proportion rating these as likely or very likely (scores 4-5) for use as compared with AI use in diagnosis or triage. The likelihood of AI use for diagnosis and triage showed lower mean scores, and triage attracted the lowest proportion of high intention, reflecting concerns about risk and medico-legal responsibility. Perceived accuracy and helpfulness among doctors followed a similar distribution: higher for drug-related and structured analytical tasks, and lower for diagnosis and triage, without statistically significant differences across task domains in the full doctor sample (p>0.05). These findings suggest that doctors generally trust AI as a supportive tool for structured decision components rather than for autonomous clinical judgement.

Nurses demonstrated consistently high mean likelihood, usefulness, accuracy and helpfulness ratings for patient monitoring (including alerts and prediction), documentation and nutrition support, with a substantial proportion rating these as “very” or “extremely” useful. In contrast, robotic nursing systems received the lowest mean scores, indicating hesitation toward automation of direct caregiving functions. Similar patterns were seen for accuracy and helpfulness, where monitoring-related items showed the highest ratings, particularly prediction of deterioration and documentation support.

Workload impact and behavioral intention

Most participants perceived that AI had the potential to be time-saving. 79.3% of doctors and 83.6% of nurses believed it would decrease workload and save time. In the converse question, the impression that artificial intelligence would increase workload was answered in the affirmative by only 9.5% of doctors and 17.1% of nurses, as shown in Table 5.

**Table 5:** : Effect of Prior AI Experience on Behavioral Intention to Use AI (All Participants). Behavioral intention items were measured using a standard 5-point Likert scale [13], consistent with UTAUT-based technology acceptance research [11,12]. Independent samples t-tests and chi-square tests were used.

Variables	Past experience with AI Score (Mean ± SD)			t-value	p-value	Past experience with AI (proportion score 4 and 5)			Chi-square value	p-value
	Yes (n=66)	No (n=190)	Overall (n=256)			Yes (n=66)	No (n=190)	Overall (n=256)		
The use of AI in my current or anticipated work environment is voluntary	3.68±1.28	3.71±1.27	3.7±1.27	-0.158	0.874	37 (56.1%)	107 (56.3%)	144 (56.3%)	0.078	0.962
I intend to use AI tools in my daily clinical activities	3.55±1.41	3.17±1.23	3.27±1.28	2.039	0.042*	41 (62.1%)	77 (40.5%)	118 (46.1%)	11.569	0.003^*^
I intend to use AI tools for clinical decision-making (e.g., diagnosis, treatment planning).	3.39±1.47	2.92±1.2	3.04±1.29	2.626	0.009*	39 (59.1%)	61 (32.1%)	100 (39.1%)	16.601	<0.001^*^
I intend to use AI tools for workflow and administrative tasks (e.g., documentation, patient monitoring).	3.85±1.28	3.72±1.16	3.75±1.19	0.748	0.455	44 (66.7%)	125 (65.8%)	169 (66%)	0.489	0.783

Perceived voluntariness of AI use was generally high: more than 60% of doctors and just over half of nurses agreed or strongly agreed that AI use in their work environment was voluntary. Behavioral intention was the strongest for workflow and administrative tasks in both professions, with nearly 70% of doctors and around 63% of nurses expressing willingness to use AI for documentation and monitoring. Intention to use AI in daily clinical activities was moderate (around 45-46% agreeing/strongly agreeing in both groups), and lowest for clinical decision-making, particularly among doctors.

Across all 256 respondents, those with prior AI experience had significantly higher intention to use AI in daily clinical activities (mean 3.55 vs. 3.17; 62.1% vs. 40.5% scoring 4-5, p=0.042 and p=0.003, respectively) and for clinical decision-making (mean 3.39 vs. 2.92; 59.1% vs. 32.1% scoring 4-5, p=0.009 and p<0.001). In contrast, intention to use AI for workflow and administrative tasks was high in both groups and did not differ significantly.

When examined across years of clinical experience, mid-career clinicians (5-15 years) in both professions were almost universally classified as early adopters using the broad definition, while strict “AI champion” status was concentrated among early-career nurses (<5 years) and a small number of mid-career doctors. However, no statistically meaningful variation in AI adoption was attributable to level of education or years of experience (all p>0.05).

**Figure 3:**
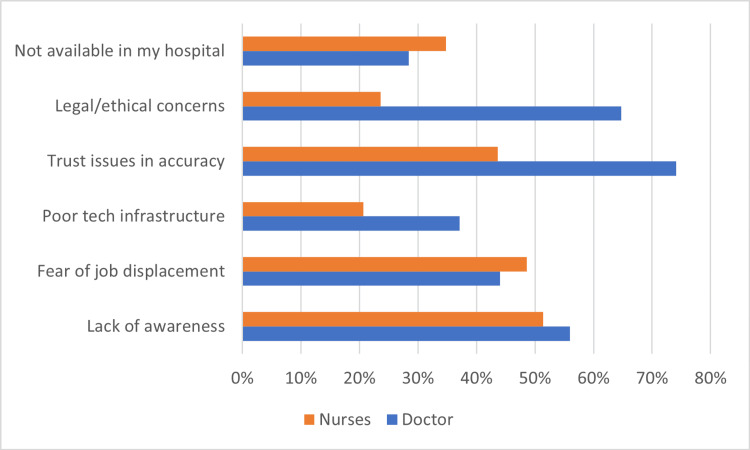
Perceived Barriers to Artificial Intelligence Adoption. Barriers were assessed using structured multiple-response items informed by UTAUT constructs, including trust, facilitating conditions, perceived risk, and awareness [11,12,13]. Not available in my hospital infers that currently no AI was operationalized and utilized in the hospital at the point in time this survey was conducted.

Perceived barriers and enablers to artificial intelligence adoption among participants are illustrated in Figure 3. Across both professions, trust issues and awareness gaps emerged as dominant barriers to AI adoption. Among doctors, 74.1% reported trust issues around accuracy and 64.7% highlighted legal/ethical concerns; among nurses, 43.6% cited trust issues, and 48.6% reported fear of job displacement. Lack of awareness was common in both groups (56% of doctors and 51.4% of nurses). While adequate technological infrastructure exists, the lack of operational AI tools in the hospital was identified as a challenge.

**Figure 4:**
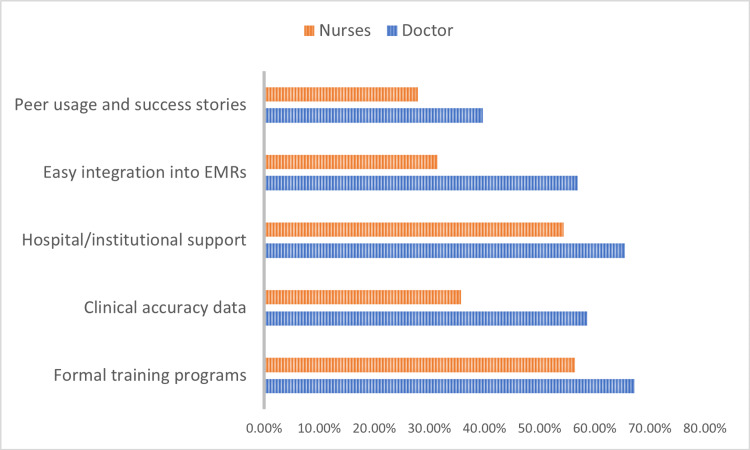
Enablers for Artificial Intelligence Adoption. Enablers reflect facilitating conditions and social influence constructs from the Unified Theory of Acceptance and Use of Technology [11,12,13].

Formal training programs were the most frequently endorsed enabler (67.2% of doctors, 56.4% of nurses), followed by hospital/institutional support and easy integration into electronic medical records. Demonstrated clinical accuracy data and peer usage or success stories were also important facilitators. These findings strongly align with the UTAUT constructs of facilitating conditions and social influence, as illustrated in Figure 4.

Early and late adopters among doctors

Predictor-Based Early Adopters

Table 6 presents the classification of early and late adopters among doctors. Among doctors, those with prior experience of artificial intelligence consistently reported higher mean scores for perceived usefulness and likelihood of adopting AI across multiple domains. Statistically significant differences were observed in the likelihood of using AI for diagnosis, which was higher among AI-experienced doctors (p=0.035), as well as in the likelihood of using AI for patient follow-up (p=0.040) and triage (p=0.041). In addition, perceived helpfulness of AI in triage was significantly higher among doctors with prior AI experience, both in terms of mean score (p=0.045) and the proportion of respondents assigning high ratings of 4 or 5 (p=0.035).

**Table 6:** Effect of Prior AI Experience on Perceived Usefulness, Accuracy, and Likelihood of AI Use Among Doctors. Likelihood, accuracy, and helpfulness items were assessed using UTAUT-based constructs measured on a 5-point Likert scale [13], adapted for healthcare artificial intelligence contexts [11,12]. Scores are presented as mean ± SD and proportion of high ratings (4-5).

Variables	Past experience with AI (mean ± SD)			t-value	p-value	Past experience with AI (proportion score 4 and 5)			Chi-square value	p-value
	Yes (n=32)	No (n=84)	Overall (n=116)			Yes (n=32)	No (n=84)	Overall (n=116)		
How likely are you to use AI in Diagnosis?	3.56±1.19	2.99±1.33	3.15±1.31	2.137	0.035*	17 (53.1%)	34 (40.5%)	51 (44%)	2.788	0.248
How likely are you to use AI for calculating drug dosing?	3.69±1.33	3.25±1.43	3.37±1.41	1.500	0.136	19 (59.4%)	42 (50%)	61 (52.6%)	2.416	0.299
How likely are you to use AI in identifying drug-drug interactions?	4.03±1.06	3.51±1.39	3.66±1.33	1.907	0.059	22 (68.8%)	48 (57.1%)	70 (60.3%)	5.222	0.073
How likely are you to use AI to help in analyzing test reports or imaging?	3.5±1.52	2.93±1.5	3.09±1.52	1.823	0.071	17 (53.1%)	35 (41.7%)	52 (44.8%)	2.127	0.345
How likely are you to use AI to help with patient follow-up?	3.84±1.02	3.31±1.31	3.46±1.25	2.081	0.040*	22 (68.8%)	39 (46.4%)	61 (52.6%)	5.319	0.070
How likely are you to use AI in triage?	3.09±1.47	2.49±1.38	2.66±1.43	2.072	0.041*	14 (43.8%)	16 (19%)	30 (25.9%)	7.508	.023^*^
How accurate do you think AI can be in diagnosing a patient?	3.03±0.97	2.8±0.98	2.86±0.98	1.153	0.251	12 (37.5%)	19 (22.6%)	31 (26.7%)	3.409	0.182
How accurate do you think AI can be in identifying appropriate drugs?	3.25±1.16	3.1±1.07	3.14±1.09	.679	0.499	14 (43.8%)	30 (35.7%)	44 (37.9%)	1.279	0.528
How accurate do you think AI can be in identifying drug interactions?	3.81±0.97	3.45±1.11	3.55±1.08	1.613	0.110	24 (75%)	46 (54.8%)	70 (60.3%)	4.567	0.102
How accurate do you think AI can be in analyzing test reports or imaging?	3.31±1.18	2.94±1.17	3.04±1.18	1.533	0.128	16 (50%)	28 (33.3%)	44 (37.9%)	3.316	0.191
SHow helpful do you think AI can be in patient follow-up?	3.75±0.88	3.4±1.07	3.5±1.03	1.632	0.105	21 (65.6%)	43 (51.2%)	64 (55.2%)	2.108	0.349
How helpful do you think AI can be in triage?	3.09±1.17	2.61±1.15	2.74±1.17	2.024	0.045*	14 (43.8%)	17 (20.2%)	31 (26.7%)	6.709	0.035^*^

Doctors with prior AI experience thus exhibit a clear early adopter profile; they are more willing to use AI for clinically consequential tasks-diagnosis, triage, and longitudinal follow-up- compared with those without such experience, who remain more cautious, especially with triage and diagnosis. Domains such as drug dosing, interaction checking, and imaging also showed higher mean scores in experienced doctors, though these differences did not reach statistical significance.

Taken together, doctors with prior AI exposure and higher ratings on core predictor items (likelihood of AI use in diagnosis, triage, and follow-up; intention to use AI in daily practice and decision-making) can be characterized as early adopters, while those without exposure, lower predictor scores, and more limited intended use form the late adopter group.

Early and late adopters among nurses

Predictor-Based Early Adopters

Among nurses, prior AI experience was associated with slightly higher mean scores and greater proportions of high ratings (4-5) across most domains, but none of these differences reached statistical significance. Despite the lack of statistically significant differences, nurses overall rated AI favorably for monitoring, documentation, and nutrition, and perceived AI as helpful in easing documentation and enhancing patient safety. The absence of strong exposure-based differences suggests that, in this group, the baseline acceptance of AI is already relatively high, and prior experience does not sharply distinguish early from late adopters in the same way as it does among doctors.

Nurses who had both formal AI/health-tech training and prior AI experience, coupled with high likelihood ratings for monitoring, documentation and nutrition, and strong intention to use AI in clinical and workflow tasks, can be regarded as early adopters from a predictor-based perspective. Those without prior AI exposure or training, and with only moderate intention scores, correspond to the late adopter segment.

**Figure 5:**
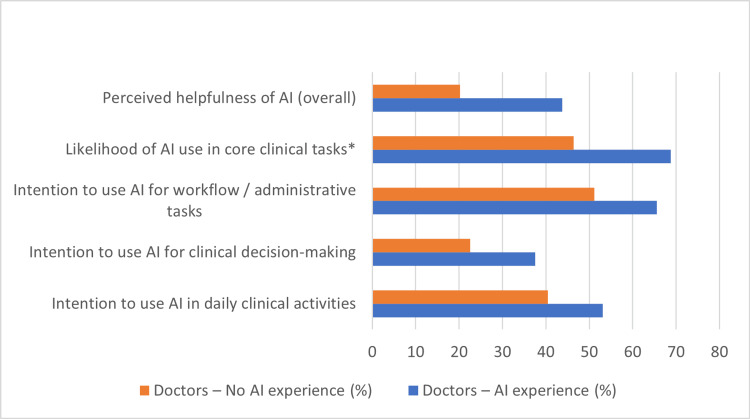
Effect of Prior AI Experience on Intention to Use and Perceived Usefulness of AI Among Doctors. Perceptions and intentions were measured using UTAUT-derived constructs, including performance expectancy and behavioral intention [11,12,13].

**Figure 6:**
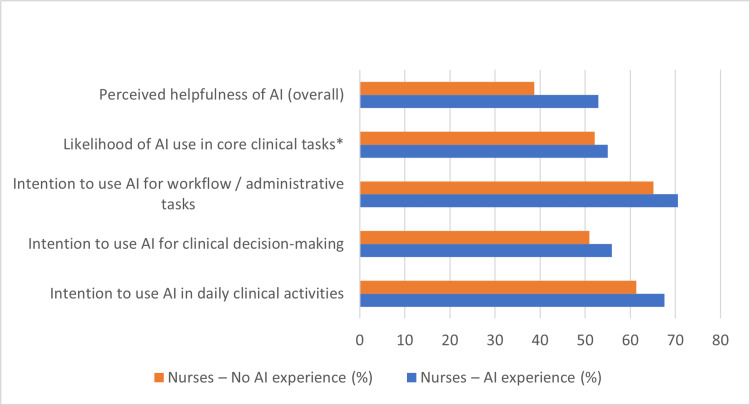
Effect of Prior AI Experience on Intention to Use and Perceived Usefulness of AI Among Nurses. Survey items were adapted from UTAUT-based technology acceptance instruments assessing perceived usefulness, likelihood of use, and behavioral intention [11,12,13].

Figure 5 presents the cross-professional comparison of early adopters among doctors, and Figure 6 presents the corresponding comparison among nurses. When early adopters were defined broadly as participants showing any marker of AI readiness (training, exposure, tool use, or high likelihood/intention), doctors and nurses appeared similarly positioned, with 92.2% and 88.6% meeting this broad readiness definition. However, under a strict definition requiring formal training, exposure, tool use and consistently high intention, only 9 individuals (3.5%) qualified as AI champions, with considerably more nurses (n=8) than doctors (n=1).

Even when early adopters were defined using a strict composite criterion (formal training, AI tool use, and consistently high likelihood and intention scores), excluding prior exposure, only 12 participants qualified (11 nurses and 1 doctor), most of whom were early-career (<5 years), indicating that strict early adoption remains rare and concentrated among junior nursing staff. This distribution was statistically significant (χ²=5.47, p=0.019), indicating that strict early adoption (excluding prior exposure) was significantly more common among nurses than doctors.

Mid-career clinicians (5-15 years) in both professions were almost universally classified as broad early adopters, while the highest density of strict early adopters among nurses was in the youngest (<5 years) group, indicating a potential generational gradient in champion-level readiness. These patterns reinforce the idea that while most healthcare professionals are open to AI in theory, structured training is required to move them from general openness to adoption.

Thematic analysis

The quantitative responses related to personal views, perceived barriers, and factors promoting AI adoption were cautiously positive but with some variation among healthcare workers. Doctors’ responses clustered into three broad perspectives. The largest group expressed strong support for AI adoption, viewing it as a valuable adjunct across multiple domains, including diagnostics (supporting clinical judgment, reducing human error, and analyzing extensive patient records), treatment (drug dosing and interaction analysis), interventions (such as laparoscopic guidance and 3D surgical planning), follow-up care (discharge summaries and AI-assisted patient query resolution), academic work (large-scale data analysis), and administrative tasks as a time-saving and efficiency-enhancing tool. AI was also perceived as a potential solution to workforce shortages. While this group was largely supportive, the need for structured training, appropriate oversight, and clarity regarding medico-legal responsibility was emphasized.

A smaller group expressed caution, highlighting that AI cannot replace the human element central to medical practice. Concerns included overdiagnosis, excessive investigations leading to resource overuse, potential erosion of clinical reasoning skills, data privacy risks, and unresolved legal issues. These respondents advocated for judicious use of AI, particularly given its early stage of development. A minority of doctors were either AI-agnostic or opposed to its use, citing the need for large-scale validation prior to implementation. Some surgeons in this group believed AI would have a limited or no role in surgical practice.

Nurses, in contrast, demonstrated largely positive attitudes toward AI, with many viewing it as a way to reduce workload, lower patient costs, and improve quality of care. However, respondents also highlighted the importance of responsible use, raising concerns related to data quality, bias, and regulatory oversight. A substantial number reported not having used AI or responded with minimal engagement (“no” or “not used”), reflecting limited exposure. Despite this, explicit distrust of AI was rare.

Nurses strongly endorsed AI for practical applications such as faster documentation during transfers or discharge, early detection of clinical deterioration, prevention of code blue events, and automated vital sign monitoring. Several respondents suggested that AI-based tools could assist patients post-discharge by addressing minor concerns through home-based applications or chatbots, thereby reducing unnecessary hospital visits. Notably, fear of job displacement was expressed among nurses but not among doctors.

Across both groups, the most common concern regarding AI was accuracy, followed by hallucinations, medico-legal uncertainty, lack of validation data, and ethical considerations. Participants emphasized that legal safeguards and integration with existing hospital systems would facilitate adoption.

For successful implementation in the Indian healthcare context, respondents stressed the need for AI systems trained on Indian datasets, affordability, multilingual capability, and its ability to function in resource-limited settings. The ability to support overburdened healthcare workers through simple, user-friendly interfaces and adaptability to diverse patient populations was considered important.

## Discussion

Our findings suggest distinct patterns of artificial intelligence readiness between doctors and nurses. Although descriptive trends suggested that mid-career clinicians (5-15 years of experience) were more consistently classified as broad early adopters, and strict early adoption was more prevalent among early-career nurses, these differences did not reach statistical significance when likelihood and intention scores were compared across years of experience. This indicates that career stage, while potentially influencing readiness through accumulated exposure or technological comfort, is not a standalone determinant, which is a pattern consistent with recent Indian studies showing that demographic variables alone do not reliably predict AI acceptance among healthcare professionals [14].

In this sample, among doctors, prior AI experience was a critical marker of adoption readiness. Those with exposure demonstrated significantly higher likelihood of using AI in high-risk domains such as diagnosis, triage, and follow-up, which carry substantial medico-legal and decision-making accountability. This reduced skepticism and increased trust echo global surveys linking prior exposure to improved AI confidence [14,15]. In contrast, doctors without experience remained cautiously positive toward structured analytical tasks (e.g., drug-drug interaction checks and dosing), consistent with evidence that clinicians trust AI more for supportive rather than autonomous diagnostic roles [16,17]. These observations align with UTAUT constructs of performance expectancy and facilitating conditions, where prior exposure and familiarity may shape perceived usefulness and intention to use technology.

Nurses, however, exhibited high baseline acceptance for monitoring, documentation, and nutrition-related tasks that were largely independent of prior AI exposure. This contrasts with some earlier studies where lack of familiarity posed a major barrier [14,18] and suggests that nurses primarily perceive AI as a workflow facilitator. This relatively uniform acceptance across exposure levels suggests that baseline perceived usefulness for workflow-related tasks may be high among nursing staff, irrespective of prior formal AI experience.

Despite this, perceived risks diverged by profession. Fear of job displacement emerged more prominently among nurses, which aligns with international surveys documenting automation anxiety [19]. Doctors, conversely, expressed greater concern over medico-legal and ethical implications in high-stakes decision-making. While trust concerns were closely linked to perceived accuracy and reliability of AI outputs, medico-legal apprehensions reflected accountability for clinical decisions supported by AI systems.

Both groups converged on key enablers: formal training, institutional support, accuracy validation, and EMR integration-core UTAUT facilitating conditions. Existing AI adoption research similarly underscores transparent validation, safety evidence, and clinical co-development as essential for building trust with AI use in healthcare [15,19]. Given the collaborative nature of patient care, interprofessional AI education programs can serve to support both the professional groups, doctors and nurses and help align workflow, and cultivate shared trust models. The strong endorsement of structured training and institutional support observed in this study indicates that facilitating conditions remain central to translating general openness toward AI into actual adoption readiness. Similar patterns of technology acceptance have been reported beyond healthcare settings, where technology readiness and social influence impact perceived usefulness and effectiveness of generative AI across professional environments, reinforcing the broader applicability of technology acceptance frameworks [20].

The strict AI champions using composite criteria that incorporate exposure, training, tool use, and uniformly high intention are unique to this study. While over 85% of participants qualified as broad early adopters, only 3.5% met strict champion criteria- predominantly early-career nurses. This gap emphasizes that general acceptance does not equate to adoption; suggesting that those with high usefulness/ accuracy/helpfulness ratings may not always translate into a high behavior intention. Structured training remains essential to translate interest into capability. Leveraging these champions as peer trainers could accelerate safe AI acceptance and gradual adoption across healthcare teams. Leveraging early adopters or AI champions as peer facilitators may support gradual and responsible integration of AI into routine clinical workflows.

Strengths

Strengths include the UTAUT-aligned design, profession-stratified comparison, and creation of both predictor-based and composite adopter classifications.

Limitations

This single-center study in a private tertiary-care hospital offers insight into early adoption dynamics; however, findings may not generalize to public-sector or resource-constrained environments. Perceptions were self-reported and may not reflect actual AI use. The cross-sectional design captures attitudes at a single time point, limiting causal inference and assessment of changes over time. A brief informational video was provided to ensure a standardized understanding of AI before assessing UTAUT parameters; however, it may have influenced perception-based scores. Likert-scale responses were analyzed descriptively without adjustment for multiple comparisons; thus, results should be interpreted as exploratory and hypothesis-generating.

## Conclusions

Doctors showed greater caution, trusting AI for structured tasks, with prior exposure significantly boosting willingness to apply it in higher-risk areas such as diagnosis, triage, and follow-up. Nurses exhibited high baseline acceptance for monitoring, documentation, and nutrition support. Shared barriers included limited awareness and trust issues, with doctors emphasizing medico-legal risks and nurses highlighting job displacement fears. Key enablers included structured training, institutional support, and EMR integration, which aligned with UTAUT facilitating conditions. These findings highlight the importance of embedding AI education within undergraduate, postgraduate, and in-service training programs rather than relying on ad hoc exposure to bridge the gap between general openness and true adoption.

## Appendices

Study questionnaire tables

Appendix A

**Table 7:** Demographic and Professional Characteristics. Items were part of a structured questionnaire adapted from UTAUT-based instruments [11,12].

Item No.	Questions	Response Type
A1	Age	Open numeric (years)
A2	Gender	Male/Female/Other
A3	Profession	Doctor/Nurse
A4	Speciality department	Open text
A5	Years of clinical experience	Open numeric
A6	Formal AI/health tech training	Yes/No
A7	Past experience with AI in healthcare	Yes/No
A8	Use of AI tools (e.g., CDSS, interaction checkers)	Yes/No
A9	Names of AI tools used	Open text

Appendix B

**Table 8:** AI Usefulness, Accuracy, and Likelihood Items for Doctors. Items were measured using a standard 5-point Likert scale following UTAUT-based constructs [11,12,13].

Item No.	Questions	Response Type
B1	Likelihood of using AI in diagnosis	5-point Likert
B2	Likelihood of using AI to identify drug appropriateness	5-point Likert
B3	Likelihood of using AI for drug dosing	5-point Likert
B4	Likelihood of using AI for drug–drug interaction checking	5-point Likert
B5	Likelihood of using AI for test report or imaging analysis	5-point Likert
B6	Likelihood of using AI for patient follow-up	5-point Likert
B7	Likelihood of using AI in triage	5-point Likert
B8	Perceived increase in workload with AI	Yes/No
B9	Perceived decrease in workload with AI	Yes/No
B10	Accuracy of AI in diagnosis	5-point Likert
B11	Accuracy of AI in identifying appropriate drugs	5-point Likert
B12	Accuracy of AI in identifying drug interactions	5-point Likert
B13	Accuracy of AI in analyzing test reports or imaging	5-point Likert
B14	Helpfulness of AI in patient follow-up	5-point Likert
B15	Helpfulness of AI in triage	5-point Likert

Appendix C

**Table 9:** AI Usefulness, Accuracy, and Likelihood Items for Nurses. Items were measured using a standard 5-point Likert scale following UTAUT-based constructs [11,12,13].

Item No.	Questions	Response Type
B16	Likelihood of using AI for vital sign alerts	5-point Likert
B17	Likelihood of using AI for prediction of deterioration	5-point Likert
B18	Likelihood of using AI for documentation	5-point Likert
B19	Likelihood of using AI for nutrition support	5-point Likert
B20	Usefulness of AI for patient mental health	5-point Likert
B21	Likelihood of using robotic nursing/caregiving systems	5-point Likert
B22	Perceived increase in workload with AI	Yes/No
B23	Perceived decrease in workload with AI	Yes/No
B24	Accuracy of AI in predicting deterioration	5-point Likert
B25	Accuracy of AI in vital sign alerts	5-point Likert
B26	Helpfulness of AI in documentation	5-point Likert
B27	Helpfulness of AI in nutrition support	5-point Likert
B28	Helpfulness of AI in mental health support	5-point Likert
B29	Helpfulness of robotic caregiving systems	5-point Likert

Appendix D

**Table 10:** Voluntariness and Behavioral Intention. Behavioral intention items were measured using UTAUT-derived constructs on a 5-point Likert scale [11,12,13].

Item No.	Questions	Response Type
C1	AI use in my work environment is voluntary	5-point Likert
C2	Intention to use AI in daily clinical activities	5-point Likert
C3	Intention to use AI for clinical decision-making	5-point Likert
C4	Intention to use AI for workflow/administrative tasks	5-point Likert

Appendix E

**Table 11:** Barriers, Enablers, and Open-ended Questions. Barriers and enablers were assessed using structured and Likert-based items informed by UTAUT constructs [11,12,13].

Item No.	Questions	Response Type
D1	Barriers to AI adoption	Multiple response
D2	Factors facilitating AI adoption	Multiple response
E1	Personal view on AI in practice	Open-ended
E2	Experience where AI helped/could help	Open-ended
E3	Concerns about AI in decision-making	Open-ended
E4	Support needed to use AI comfortably	Open-ended
E5	AI characteristics needed for Indian healthcare	Open-ended
